# Comparison of thiazide‐like diuretics *versus* thiazide‐type diuretics: a meta‐analysis

**DOI:** 10.1111/jcmm.13205

**Published:** 2017-06-19

**Authors:** Wenjing Liang, Hui Ma, Luxi Cao, Wenjiang Yan, Jingjing Yang

**Affiliations:** ^1^ The Key Laboratory of Cardiovascular Remodeling and Function Research Chinese Ministry of Education and Chinese Ministry of Health and The State and Shandong Province Joint Key Laboratory of Translational Cardiovascular Medicine Qilu Hospital of Shandong University Jinan Shandong China; ^2^ Department of Pediatrics and Department of Cardiology Shandong Provincial Hospital Affiliated to Shandong University Jinan Shandong, China; ^3^ Kidney Disease Center First Affiliated Hospital School of Medicine Zhejiang University Hangzhou Zhejiang China

**Keywords:** thiazide‐like diuretics, thiazide‐type diuretics, hypertension, hypokalemia, hyponatremia

## Abstract

Thiazide diuretics are widely used for the management of hypertension. In recent years, it has been actively debated that there is interchangeability of thiazide‐type diuretics hydrochlorothiazide and thiazide‐like diuretics including indapamide and chlorthalidone for the treatment of hypertension. With the purpose of seeking out the best thiazide diuretic for clinicians, we summarized the existing evidence on the two types of drugs and conducted a meta‐analysis on their efficacy in lowering blood pressure and effects on blood electrolyte, glucose and total cholesterol. Twelve trials were identified: five based on the comparison of indapamide *versus* hydrochlorothiazide and seven based on the chlorthalidone *versus* hydrochlorothiazide. In the meta‐analysis of blood pressure reduction, thiazide‐like diuretics seemed to further reduce systolic BP ([95% CI]; −5.59 [−5.69, −5.49]; *P* < 0.001) and diastolic BP ([95% CI]; −1.98 [−3.29, −0.66]; *P* = 0.003). Meanwhile, in the analysis of side effects, the incidence of hypokalemia ([95% CI]; 1.58 [0.80, 3.12]; *P* = 0.19), hyponatremia ([95% CI]; −0.14 [−0.57, 0.30], *P* = 0.54), change of blood glucose ([95% CI];0.13 [−0.16, 0.41], *P* = 0.39) and total cholesterol ([95% CI]; 0.13 [−0.16, 0.41], *P* = 0.39) showed that there is no statistical significant differences between the two groups of drugs. In conclusion, using thiazide‐like diuretics is superior to thiazide‐type diuretics in reducing blood pressure without increasing the incidence of hypokalemia, hyponatraemia and any change of blood glucose and serum total cholesterol.

## Introduction

Thiazide diuretics were once the first effective oral antihypertensive agents with an acceptable side‐effect profile. For more than a half‐century, thiazide diuretics have been used for the management of hypertension 1. Despite structural variation among the heterogeneous group of agents including the thiazide‐type as well as thiazide‐like diuretics, the term ‘thiazide diuretic’ incorporates all diuretics believed to have a primary action in the distal tubule 1. The publication of 2014 ‘Evidence‐Based Guideline for the Management of High Blood Pressure in Adults’ from the Panel Members Appointed to the Eighth Joint National Committee (JNC 8) recommended the ‘thiazide‐type diuretics’ as first‐line therapy 2. In JNC 8, the recommendation is specific for diuretics, which includes thiazide‐like diuretics including indapamide and chlorthalidone, and it does not include loop or potassium‐sparing diuretics 3. Based on the evidence of some large‐scale randomized clinical trials (RCTs), the status of thiazide diuretics keeps playing a more vital role in treating hypertension.

Recently, the interchangeability of hydrochlorothiazide and chlorthalidone has been a matter of intense debate 4. After single oral doses, HCTZ achieved peak concentrations in ≈2 hr and had a half‐life of ≈6.5 to 9 hr. The half‐life of HCTZ would suggest that the drug should be given twice daily. Compared to HCTZ, chlorthalidone has a longer half‐life, almost 42 hr (range, 29–55 hr). At the same time, inspection of the available studies suggests that 50 mg HCTZ is approximately equivalent to 25–37 mg chlorthalidone. In other words, it suggests that equivalent doses of chlorthalidone should generally be 50–75% of typical HCTZ doses 5. Both HCTZ and chlorthalidone have demonstrated cardiovascular events risk reduction in clinical trials. However, the largest trials, including, The Systolic Hypertension in the Elderly Program (SHEP) study 6, 7, elaborated that only chlorthalidone can significantly lower rates of stroke as well as some other fatal or non‐fatal cardiovascular events. The Antihypertensive and Lipid Lowering Treatment to Prevent Heart Attack (ALLHAT) study 8 that included over 30,000 hypertensive patients compared chlorthalidone to alpha‐blockers, calcium channel blockers and ACE inhibitors. This randomized controlled trial demonstrated that at 12.5 and 25 mg/day dosages, chlorthalidone was beneficial in reducing new‐onset heart failure compared with the other treatment used in the trial. Although there were strong evidence supporting the idea that chlorthalidone should be recommended as the initial treatment of hypertension, a study comparing these two types of diuretics directly is missing.

Indapamide, a member of thiazide‐like agents, presents some differences with others. In fact, besides its diuretic properties, indapamide presents some protective effects on vascular or organ damage in animal and human investigations. In some clinical trials, it was effective in reducing left‐ventricular mass index in hypertensive patients and remarkably improving the renal function 9. Furthermore, in a recent large‐scale RCT, use of a sustained‐release indapamide‐based regimen as initial therapy led to relative reductions of 39%, 64% and 21% in the rates of fatal stroke, heart failure and death, respectively, in patients older than 80 years of age 10. Although much evidence demonstrated the effectiveness and advantages of indapamide, there is no large‐scale immediate clinical trial and meta‐analysis to evaluate the BP lowering efficacy and safety between indapamide and HCTZ.

To examine whether thiazide‐like diuretics were superior over the thiazide‐type diuretics in lowering blood pressure without affecting the biochemical properties, we undertook a meta‐analysis of clinical trials with either HCTZ *versus* chlorthalidone or HCTZ *versus* indapamide as the independent comparative arms.

## Methods

### Data sources and search strategy

In our meta‐analysis, we adhered to the PRISMA guideline strictly. The search strategy was designed by the first author and reviewed by another two authors. We searched for evidence in PubMed (1948–2017/Jan) and Embase (1980–2017/Jan), Google Scholar, the ScienceDirect, the metaRegister of Controlled Trials and the Cochrane Central Register of Controlled Trials using the following terms: (indapamide OR chlorthalidone) AND (hydrochlorothiazide) AND(systolic blood pressure OR diastolic blood pressure)AND (cardiovascular events OR antihypertensive efficacy OR heart rates OR electrolyte disturbance OR hypokalemia OR hyponatremia OR serum cholesterol OR blood glucose OR hyperglycemia OR hypercholesteremia OR adverse effects). Moreover, references about the relevant reviews were also sought.

### Inclusion and exclusion criteria

Trial inclusion criteria met the following criteria: (1) randomized clinical trial or double‐blind controlled trial of thiazide‐like or thiazide‐type therapy in people with hypertension (SBP ≥ 140 mmHg or DBP ≥ 90 mmHg); (2) patients were allocated to 2 monotherapy thiazide or combined with other kind of antihypertensive drugs in fixed‐dose arms; (3) duration of follow‐up ≥4 weeks; (4) baseline washout and run‐in phase of medication ≥1 week; (5) measurements of ≥1 of the following, systolic BP, diastolic BP, serum potassium, uric acid, serum cholesterol, glucose or heart rate. There were not yet enough trials including thiazide‐like or thiazide‐type diuretics for meta‐analysis with the exception of hydrochlorothiazide, chlorthalidone and indapamide, so only trials including the comparison between hydrochlorothiazide and chlorthalidone or hydrochlorothiazide and indapamide were included. We excluded trials where subjects were predefined as responders or non‐responders before the trial. If the study used the titration method in step‐up protocols, we just extract the data using the initial doses. Studies using potassium supplementation were included, but step‐down and drug withdrawal protocols were ineligible. Trials were also ineligible if participants were <18 years old or had cirrhosis with ascites, type 1 or 2 diabetes, nephrotic syndrome, renal insufficiency, documented serum creatinine level >1.5 times normal, history of ischaemic stroke, unstable angina or myocardial infarction within the past 6 months, cardiac failure, secondary hypertension, dementia or other cognitive impairment, pregnancy or lactating women. If the study condition permits, we used the blood pressure records *via* ambulatory blood pressure monitoring, if not, where resting BP measurements were available for >1 time during a 24‐hr period, the trough measurement, defined as 22–26 hr after dose, was used. When BP was recorded in multiple positions, sitting BP was used, unless variance data were only given for another position, in which case that position was used.

### Study characteristics and quality assessment

The characteristics of studies 11, 12, 13, 14, 15, 16, 17, 18, 19, 20, 21, 22 used for the meta‐analysis were shown in Table 1. These studies were published from 1983 to 2016. A total number of 1580 patients from 12 trials were studied, in which 10 and 11 studies provided the data of systolic BP and diastolic reduction after treatment, respectively. In the thiazide‐like diuretics group, we integrated the indapamide and chlorthalidone together. As there was lack of the head‐to‐head comparison trials between chlorthalidone and HCTZ, we found only three eligible trials used them in the monotherapy arm, while the remaining four trials compared the chlorthalidone and HCTZ both combined with another drug. All of the included studies were published in English. Score developed from the criteria of Jadad was utilized to assess study quality 23, which had a possible range from zero to five, including double blinding, randomization and drop‐outs. It was defined as high quality if a study scored range from three points to five points.

**Table 1:** Baseline characteristics of the included studies; CTD: chlorthalidone; HCTZ: hydrochlorothiazide; IND: indapamide; IND SR: indapamide sustained release tablet; metoprolol; XL: metoprolol extended release; AZL‐M/CLD: azilsartan medoxomil/chlorthalidone single‐pill combination; AZL‐M + HCTZ: azilsartan medoxomil + hydrochlorothiazide co‐administered

Study (author, year)	BP inclusion criteria	Quality score	Subjects (*N*)	Interventions	Duration of washout and run‐in (weeks)	Duration of treatment (weeks)	Results measurement
Radevski *et al*. (2002)	Sitting DBP 95‐115 mmHg	3	42	12.5 mg HCTZ *vs*. 2.5 mg IND	3	12	24‐hr ABPM
David *et al*. (1999)	Sitting DBP 95–105 mmHg	3	39	25 mg HCTZ *vs*. 2.5 mg IND	4	24	Supine BP
GERARD *et al*. (1983)	Sitting DBP ≥ 95 mmHg	3	24	50 mg HCTZ *vs*. 2.5 mg IND	6	12	Recumbent BP
A.Pareek *et al*. (2009)	SBP 140–179 mmHg or DBP 90–109 mmHg	3	60	50 mg Metoprolol XL + 6.25 mg CTD *vs*. 50 mg Metoprolol XL + 12.5 mg HCTZ;	1	8	Seated BP; plasma potassium record
Leonetti *et al*. (2004)	Supine DBP 95–114 mmHg Supine SBP 161–209 mmHg	4	354	25 mg HCTZ *vs*. 1.5 mg Indapamide SR	4	12	Supine BP
Kwon *et al*. (2012)	Never treated HTN(SBP ≥ 140 mmHg and/or DBP ≥ 90 mmHg)	3	28	8 mg Candesartan + 25 mg HCTZ *vs*. 8 mg Candesartan + 12.5 mg CTD	4	8	Supine brachia BP
Michael *et al*. (2006)	SBP 140–179 mmHg or DBP 90–109 mmHg	4	24	Firstly: 12.5 mg CTD *vs*. 25 mg HCTZ; at week 4: force‐titrated to 25 mg CTD *vs*. 50 mg HCTZ	4	8	24‐hr ABPM
Pareek *et al*. (2009)	SBP 140–179 mmHg or DBP 90–109 mmHg	3	120	25 mg Losartan + 6.25 mg CTD *vs*. 25 mg Losartan + 12.5 mg HCTZ	2	4	Office blood pressure measurement
Senior *et al*. (1993)	DBP 95–120 mmHg	4	40	25 mg HCTZ *vs*. 2.5 mg IND	2	24	Diastolic blood pressure
Siegel *et al*. (1992)	DBP 90–105 mmHg	5	233	50 mg HCTZ *vs*. 50 mg CTD	4	8	24‐hr Holter monitoring and laboratory tests
Bakris *et al*. (2012)	Seated SBP 160–190 mmHg	4	587	40 mg AZL‐M/12.5 mg CTD *vs*. 40 mg AZL‐M + 12.5 mg HCTZ	4	4	24‐hr mean BP
Anil K. Pareek *et al*. (2016)	Office SBP between 140 and 159 mmHg and DBP between 90 and 99 mmHg	5	34	12.5 mg HCTZ *vs*. 6.25 mg CTD	2	12	24‐hr ambulatory blood pressure monitoring

### Data extraction and statistical analysis

The primary end‐point for our analysis was the systolic and/or diastolic BP reduction, the incidence of hypokalemia, hyponatremia and the change of serum total cholesterol and glucose throughout the different drug therapy. The changes of blood pressure, serum TG and glucose were computed as the difference in the BP values at the final follow‐up (or specific time‐point if multiple time‐points were provided) compared to the baseline or initial measurement. However, we analyse the incidence of hypokalemia using the dichotomous method. All data extracted from the 12 studies were recorded in Microsoft Excel before transfer to Review Manager version 5.0 program for analysis. Continuous variables were conveyed in the form of the mean and standard deviation (SD). Research results data whose means and SDs were unavailable were kicked out of the meta‐analysis. Heterogeneity was calculated using the I‐square statistic 24. I‐squared is the ratio of true heterogeneity to total variation in observed effect, and it will not be impacted by the size of studies. All analyses were initially carried out using a fixed‐effects model. However, if heterogeneity across studies was observed, the analyses were carried out with a random‐effects model. The random‐effects model of inverse variance was used to calculate the odds ratio (OR) with 95% confidence interval (95% CI).

## Results

### Characteristics of studies

**Figure 1:**
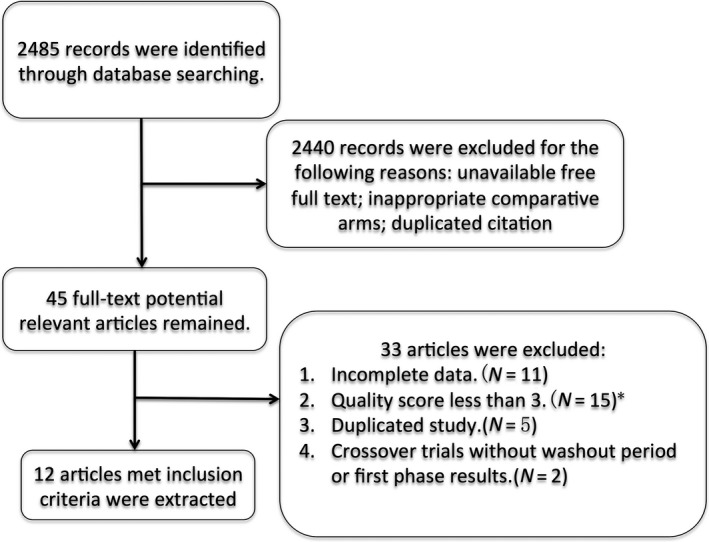
Screening and study selection process. * Study quality was judged by the Jadad score.

A total of 2485 records were found after searching in PubMed, Embase, Google Scholar and other databases. As some literatures did not provide free full texts, or the inappropriate comparative methods, only 45 full‐text articles were reviewed after the first selection process (Fig. 1). In the second selection process, we have filtered out 33 articles, which do not contain complete available data; lower than 3 when doing quality score assessment according to Jadad score; do not have washout period. Ultimately, twelve studies were maintained in our re‐analysis. The characteristic of included studies was described in this article (Table 1).

### SBP reduction

**Figure 2:**
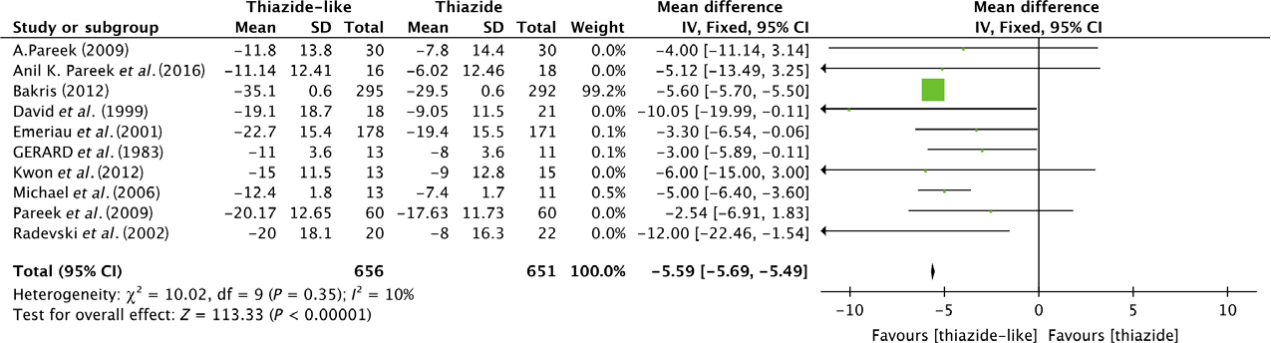
Forest plot shows the variance of systolic BP reduction by thiazide‐like diuretics *versus* thiazide diuretics.

In all of the included studies, 1307 patients from 10 trials provided data of systolic BP change from the baseline to the end‐point of treatment period. Figure 2 shows the forest plot of different lowering systolic blood pressure efficacy between thiazide‐like and thiazide diuretics. The final merged results show that thiazide‐like diuretics (indapamide or chlorthalidone) will be significantly effective in lowering systolic BP(pooled effect size [95% CI]; −5.59 [−5.69, −5.49]; Heterogeneity: Chi² = 10.02, df = 9 (*P* = 0.35); I² = 10%) than thiazide‐type diuretics (hydrochlorothiazide).

### DBP reduction

**Figure 3:**
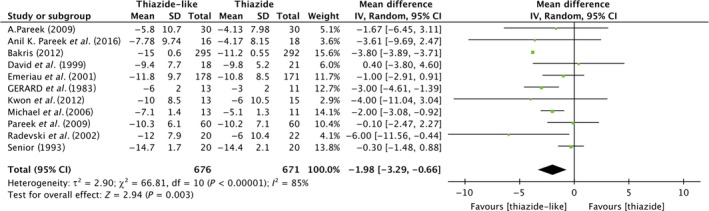
Forest plot shows the variance of diastolic BP reduction by thiazide‐like diuretics *versus* thiazide diuretics.

A meta‐analysis of diastolic BP reductions between the thiazide‐like and thiazide‐type diuretics also showed a statistically meaningful result. As shown in Figure 3, among 1347 subjects from 11 trials, compared to hydrochlorothiazide, indapamide or chlorthalidone, can lower diastolic blood pressure more effectively (pooled effect size [95% CI]; −1.98 [−3.29, −0.66]; Heterogeneity: Tau² = 2.90; Chi² = 66.81, df = 10 (P < 0.00001); I² = 85%). The heterogeneity exists.

### Incidence of hypokalemia

**Figure 4:**
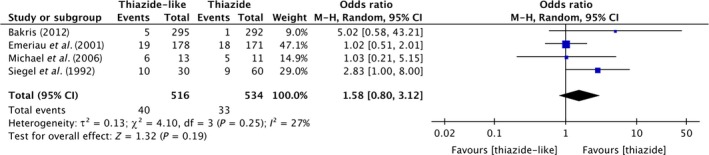
Forest plot shows the incidence of hypokalemia using thiazide‐like diuretics *versus* thiazide diuretics.

Four trials provided data of incidence of hypokalemia happened during or after the treatment period among 1050 subjects. It is shown in Figure 4, using thiazide‐like diuretics indapamide or chlorthalidone, patients will have the same risks of hypokalemia with hydrochlorothiazide users (pooled effect size [95% CI]; 1.58 [0.80, 3.12], *P* = 0.16; Heterogeneity: Tau² = 0.13; Chi² = 4.10, df = 3 (*P* = 0.25); I² = 27%).

### Incidence of hyponatremia

**Figure 5:**
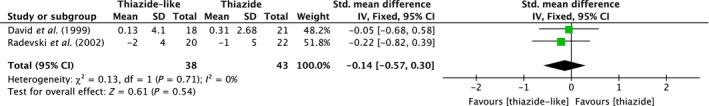
Forest plot shows the incidence of hyponatremia using thiazide‐like diuretics *versus* thiazide diuretics.

As shown in Figure 5, data from two trials showed the same risk of hyponatremia in users of thiazide‐like group and thiazide‐type group (pooled effect size [95% CI]; −0.14 [−0.57, 0.30], *P* = 0.54; Heterogeneity: Chi² = 0.13, df = 1 (*P* = 0.71); I² = 0%).

### Total cholesterol and glucose

**Figure 6:**
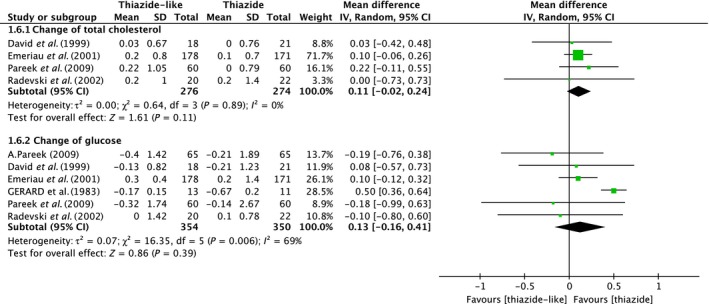
Forest plot shows the change of serum total cholesterol and glucose after using the drug therapy.

Four trials included data from 550 subjects; as shown in Figure 6, there were no statistically significant differences of total cholesterol between the two treatment groups (pooled effect size [95% CI]; 0.11 [−0.02, 0.24], *P* = 0.11; Heterogeneity: Tau² = 0.00; Chi² = 0.64, df = 3 (*P* = 0.89); I² = 0%). In the meta‐analysis of change of serum glucose, we scanned seven trials included 804 subjects and the results also show no significant differences between thiazide‐like and thiazide‐type diuretics (pooled effect size [95% CI]; 0.13 [−0.16, 0.41], *P* = 0.39; Heterogeneity: Tau² = 0.07; Chi² = 16.35, df = 5 (*P* = 0.006); I² = 69%). The heterogeneity exists.

### Heterogeneity and sensitivity analyses

We can believe that there is no heterogeneity exists in the analysis of systolic blood pressure reduction, incidence of potassium and total cholesterol because mild heterogeneity might account for I^2^ is <30% of the variability in point estimates. However, in the meta‐analysis of diastolic blood pressure reduction and the change of serum glucose, a notable heterogeneity is observed as I^2^ is more than 50% 23. To solve the problem, we have carried out the sensitivity analyses to exclude lower‐quality studies, but there is no change of the results even when used another statistical model (data not shown).

## Discussion

For meta‐analysis, we searched for published thiazide diuretic‐related studies that are three decades old. We extracted useful data from 12 included clinical trials. We reanalysed them and made a series of outcomes. The potent of thiazide‐type and thiazide‐like diuretics differed quite remarkably. We found out that thiazide‐like diuretics, indapamide and chlorthalidone, have an advantage in lowering both systolic and diastolic blood pressure without significantly increasing the risks of hypokalemia and hyponatremia, or making a significant change of blood glucose and serum total cholesterol compared to the most prescribed thiazide‐type diuretics such as hydrochlorothiazide.

Thiazide diuretics are the second most common type of antihypertensive drug. Therefore, it continues to be widely used in the treatment of hypertension. Whereas in the past half‐century, there was few studies which compared the thiazide‐type and thiazide‐like diuretics directly in random trials. To provide more powerful evidence for clinical therapy, it is essential to make a comprehensive comparison between the most common prescribed antihypertensive agents. In consideration of the side effects of thiazide diuretics, the major concern might be electrolyte disturbance, serum glucose and total cholesterol. Due to the longer half‐life of thiazide‐like diuretics, risks of adverse events would be expected to increase, particularly the sodium and potassium homoeostasis disorders. Our analysis made a comprehensive comparison of thiazide‐type and thiazide‐like diuretics to indicate that to achieve the same level of BP reduction, thiazide‐like diuretics were not worse than thiazide‐type diuretics in increasing risks of electrolyte disturbance.

Antihypertensive drugs are not only able to lower blood pressure, but there are many extra benefits in the cardiovascular system, such as anti‐inflammatory, anti‐atherosclerosis, improve cardiac function and target organ protection. Several clinical studies had shown that low‐dose diuretics could exert target organ protection effect when compared to other antihypertensive 25, 26.

Our findings of this study should be set in context of previous meta‐analyses. In a dose‐stratified meta‐analysis and metaregression of 26 studies, Peterzan et al. 27 characterized the dose–response relationships for three commonly prescribed thiazide/thiazide‐like diuretics, hydrochlorothiazide, chlorthalidone and bendroflumethiazide. He showed that chlorthalidone is more effective than hydrochlorothiazide both for BP reduction and biochemical outcomes. He also provided us the recommended dose and dose–effect relationship of these diuretics. In another large‐scale meta‐analysis published last year 28, thiazide‐like diuretic could reduce risk of cardiovascular events and heart failure compared to thiazide‐type diuretics. In this meta‐analysis, the author used both placebo and other antihypertensive drugs as control arm, which would probably induce a biased baseline of the comparison. There was no detailed adverse effect analysis in this article. To avoid the difference in baseline, we only included studies that used both thiazide‐like and thiazide‐type diuretics as different arm but in the same trial. Additionally, this is the first meta‐analysis, which highlighted the comparison of the major adverse effects of these two diuretics.

Electrolyte disturbance might be a major concern for prescribing thiazide diuretic. Jan C. performed a case–control study 29, which found an increase risk of hyponatremia in CTDN arm compared to HCTZ arm when given the equal dose, whereas there is no significantly increased incidence of hyponatremia in using CTDN compared with twice the dose of HCTZ per day (CTDN 12.5 mg/day *vs*. HCTZ 25 mg/day and CTDN 25 mg/day *vs*. HCTZ 50 mg/day). The dose‐titrate of CTDN and HCTZ was mentioned in Berkris *et al*. study 11. Therefore, a lower dose of CTDN required to achieve the same BP reduction as well as cardiovascular outcomes might also reduce the risk of hyponatremia. Likewise, in our study, we also performed the same results that using thiazide‐like diuretic would not increase the incidence of hyponatremia compared to HCTZ treatment.

Several limitations exist in our meta‐analysis. First, in two of the included studies, the original design is a crossover study, but to insure statistical robustness of the data in the presence of a possible carryover effect, we decided that only the data from those who complete the first active treatment period would be analysed. Secondly, we combined the indapamide and chlorthalidone together as one of the comparison arm without considering the variance between these two agents. On the other hand, we did not do the dose–response of the three drugs separately. This therefore means that we cannot neglect the error from different doses in the same group. Thirdly, some of our included studies were published long ago with the differences in inclusion and exclusion criteria, BP measurement techniques and drug formulation. Although the score of these trials (as assessed by Jadad criteria) is more than 3, the data from these studies will contribute to heterogeneity between studies. As there is lack of head‐to‐head comparative trials between chlorthalidone and HCTZ, we used the data from a combination of them with beta‐blocker, ACEI or calcium channel blocker. The combination trials might make it hard to access the effects on blood pressure reduction efficacy, the risk of incidence of hypokalemia and hyponatremia and the change of blood glucose and total cholesterol of thiazide diuretics, which would make the results of the meta‐analysis less compelling.

In summary, we performed a meta‐analysis for the controversial clinical decision that whether thiazide‐type or thiazide‐like diuretics should be more prescribed as the initial antihypertensive therapy. Our analysis obtained the different efficacy of BP reduction and biochemical outcomes when used the two kinds of drugs. It is recommended that further randomized controlled trials should be done to compare these two types of drugs. The available conclusion from our analysis suggests that thiazide‐type diuretic should be replaced by the thiazide‐like diuretic, which possess higher BP reduction efficacy and no more risk of electrolyte disturbance and metabolic disorders.

## Conflict of interests

No potential conflict of interests were disclosed.

## Authors’ contributions

W.J.L designed and conducted the analysis and wrote the manuscript. H.M and L.X.C collected and evaluated the data. J.J.Y and W.J.Y contribute to the design, analysis and revised the manuscript. J.J.Y is the guarantor of the work and takes the responsibility and accuracy for the integrity of the results.
